# Ontogeny of kainate-induced gamma oscillations in the rat CA3 hippocampus *in vitro*

**DOI:** 10.3389/fncel.2015.00195

**Published:** 2015-05-20

**Authors:** Vera Tsintsadze, Marat Minlebaev, Dimitry Suchkov, Mark O. Cunningham, Roustem Khazipov

**Affiliations:** ^1^INMED, INSERM U-901Marseille, France; ^2^Aix-Marseille UniversityMarseille, France; ^3^Laboratory of Neurobiology, Kazan Federal UniversityKazan, Russia; ^4^Institute of Neuroscience, The Medical School, Newcastle UniversityNewcastle upon Tyne, UK

**Keywords:** kainate, gamma, beta, oscillations, CA3 region, hippocampal, gaba receptors, NKCC1

## Abstract

GABAergic inhibition, which is instrumental in the generation of hippocampal gamma oscillations, undergoes significant changes during development. However, the development of hippocampal gamma oscillations remains largely unknown. Here, we explored the developmental features of kainate-induced oscillations (KA-Os) in CA3 region of rat hippocampal slices. Up to postnatal day P5, the bath application of kainate failed to evoke any detectable oscillations. KA-Os emerged by the end of the first postnatal week; these were initially weak, slow (20–25 Hz, beta range) and were poorly synchronized with CA3 units and synaptic currents. Local field potential (LFP) power, synchronization of units and frequency of KA-Os increased during the second postnatal week to attain gamma (30–40 Hz) frequency by P15–21. Both beta and gamma KA-Os are characterized by alternating sinks and sources in the pyramidal cell layer, likely generated by summation of the action potential—associated currents and GABAergic synaptic currents, respectively. Blockade of GABA(A) receptors with gabazine completely suppressed KA-Os at all ages indicating that GABAergic mechanisms are instrumental in their generation. Bumetanide, a NKCC1 chloride co-transporter antagonist which renders GABAergic responses inhibitory in the immature hippocampal neurons, failed to induce KA-Os at P2–4 indicating that the absence of KA-Os in neonates is not due to depolarizing actions of GABA. The linear developmental profile, electrographic features and pharmacological properties indicate that CA3 hippocampal beta and gamma KA-Os are fundamentally similar in their generative mechanisms and their delayed onset and developmental changes likely reflect the development of perisomatic GABAergic inhibition.

## Introduction

Neuronal synchronization in gamma (30–80 Hz) oscillations is fundamental for a variety of higher order cortical processes (Gray and Singer, 1989; Singer and Gray, 1995; Fries et al., 2001; Buzsáki and Draguhn, 2004; Fries, 2009; Wang, 2010; Whittington et al., 2011; Buzsáki and Wang, 2012). Considerable evidence indicates that the synchronization of neurons in gamma oscillation is based on synchronous inhibition through fast-spiking perisomatic parvalbumin-containing basket interneurons (Buhl et al., 1998; Fisahn et al., 1998; Bartos et al., 2007; Mann and Paulsen, 2007; Whittington et al., 2011; Buzsáki and Wang, 2012). Inhibition-based gamma oscillations can be reliably induced in hippocampal and neocortical slices *in vitro* and *in vivo* during exposure to cholinergic agents or glutamate receptor agonists including kainate (Fisahn et al., 1998, 2004; Hájos et al., 2000; Hormuzdi et al., 2001; Csicsvari et al., 2003; Khazipov and Holmes, 2003; Pais et al., 2003; Mann et al., 2005; Sakatani et al., 2008; Gulyás et al., 2010; Oren et al., 2010; Haggerty et al., 2013).

Inhibition-based gamma oscillations emerge relatively late in development. In humans, gamma oscillations emerge several months after birth and show a developmental increase until adulthood (for review, Uhlhaas et al., 2010). Similarly, in rodents *in vivo*, hippocampal gamma oscillations emerge by the end of first postnatal week and they are initially organized in short-living bursts at 20–30 Hz (Dzhala et al., 2001; Lahtinen et al., 2002; Leinekugel et al., 2002; Doischer et al., 2008; Mohns and Blumberg, 2008). This delayed development of gamma oscillations has been suggested to reflect the delayed maturation of the perisomatic inhibition. Indeed, in rodents, immature cortical neurons display elevated intracellular chloride concentration and GABA exerts depolarizing and excitatory action on them during the first postnatal week (Ben-Ari et al., 1989, 2007; Khazipov et al., 2015), including perisomatic compartment of CA3 pyramidal cells (Tyzio et al., 2006, 2008; Doischer et al., 2008; Minlebaev et al., 2013) and interneurons (Khazipov et al., 1997; Doischer et al., 2008; Tyzio et al., 2008; Sauer and Bartos, 2010). In addition, basket cells develop fast spiking features, form synapses with excitatory cells and establish chemical and electrical synapses with other basket cells only towards the end of the first postnatal week, and the maturation of these cells proceeds through the first postnatal month (Du et al., 1996; Tyzio et al., 1999; Chattopadhyaya et al., 2004; Daw et al., 2007; Huang et al., 2007; Doischer et al., 2008; Okaty et al., 2009; Wang and Gao, 2010; Goldberg et al., 2011; Pangratz-Fuehrer and Hestrin, 2011; Yang et al., 2014). A developmental model of gamma rhythmogenesis based on the developmental changes in perisomatic inhibition predicts that gamma oscillations should emerge in the rodent hippocampus by the end of the first postnatal week, and that they should be initially slow and poorly coherent (Doischer et al., 2008). In the present study, we addressed the developmental features of gamma oscillations by exploring a model of kainate-evoked gamma oscillations in the CA3 region of the rat hippocampal slices.

## Materials and Methods

### Ethical Approval

All animal-use protocols followed the guidelines of the French National Institute of Health and Medical Research (INSERM, protocol N007.08.01) and the Kazan Federal University on the use of laboratory animals (ethical approval by the Institutional Animal Care and Use Committee of Kazan State Medical University N9–2013).

### Brain Slice Preparation

**Figure 1:**
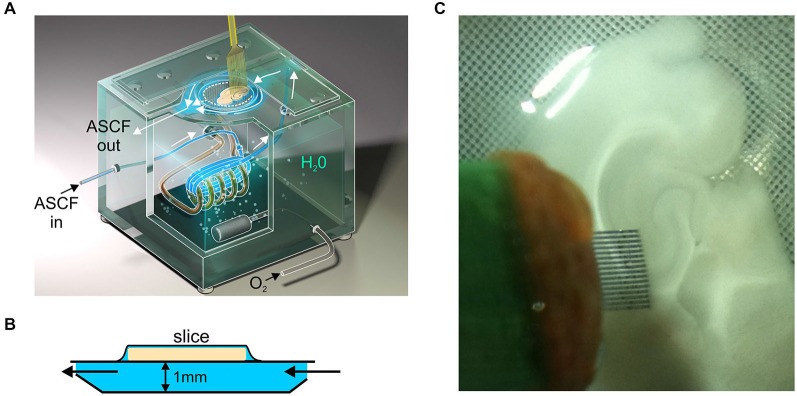
**Scheme of recordings in the modified interface chamber. (A)** Orthogonal scheme of the modified interface chamber. Slice is placed on the plastic mesh and its inner surface is superfused with ACSF to improve oxygenation at the bottom of slice and to accelerate drug delivery. **(B)** Scheme of the modified interface chamber, side view. Note 1 mm high perfusion space underneath the slice. **(C)** Photograph of recordings with 16-shank silicone probe from the hippocampal slices in the modified interface chamber.

Acute coronal brain slices were prepared from P2–21 Wistar rats. P0 was the day of birth. The animals were anesthetized with isoflurane, decapitated and the brain was rapidly removed to oxygenated (95% O_2_–5% CO_2_) ice-cold (2–5°C) artificial cerebrospinal fluid (ACSF) of the following composition (in mM): NaCl 126, KCl 3.5, CaCl_2_ 2, MgCl_2_ 1.3, NaHCO_3_ 25, NaH_2_PO_4_ 1.2 and glucose 11 (pH 7.4). Four hundred μm thick coronal slices were cut using a Vibratome (VT 1000E; Leica, Nussloch, Germany). Slices were kept in oxygenated ACSF at room temperature (20–22°C) for at least 1 h before use. For recordings slices were placed into a modified interface chamber on the plastic mesh with 1 mm high perfusion space underneath the mesh (Figure 1) and superfused on the inner side with oxygenated ACSF at 33.5–34°C at a flow rate of 2–4 ml/min.

### Electrophysiological Recordings

Extracellular recordings of the local field potentials (LFP) and multiple unit activity (MUA) were performed from the CA3 region of hippocampus using Menendez de la Prida’s sixteen shank silicon probes with 100 μm separation distance between the shanks (Figure 1B) or 4 × 4 16 channel probes with four tetrodes horizontally separated by 200 μm (Neuronexus Technologies, USA). The signals from extracellular recordings were amplified and filtered (10,000× 0.15 Hz–10 kHz) using a 32-channel amplifier (DIPSI, France), digitized at 40 kHz and saved on a PC for *post hoc* analysis.

Patch-clamp recordings were performed from CA3 pyramidal cells located in vicinity of the extracellular electrodse (separation distance <100 μm) using Axopatch 200B amplifier (Axon Instruments, Union City, CA, USA) Patch electrodes were made from borosilicate glass capillaries (GC150F-15, Clark Electromedical Instruments) and had a resistance of 4–7 MOhm. Whole cell recordings were performed using pipette solutions of the following composition (mM): 130 K-gluconate, 10 Na-gluconate 4 (P14–15) or 14 (P5–6) NaCl, 4 MgATP, 4 phosphocreatine, 0.3 GTP, and 10 HEPES, pH 7.25; osmolarity 270–280 mOsm. Recordings of the glutamate receptor mediated excitatory postsynaptic currents (EPSCs) were performed at the reversal potential of the GABA(A) receptor mediated IPSCs and IPSCs were recorded at the reversal potential of EPSCs.

### Data Analysis

Raw data were preprocessed using custom-written functions in MATLAB (MathWorks, USA). Briefly, raw data were explored to detect MUA, following which the raw data were down-sampled to 1000 Hz. MUA was detected at a band-passed signal (>400 Hz and <4000 Hz), where all negative events exceeding 3.5 standard deviations were considered to be spikes. Further analysis of extracellular units and LFP data were performed using custom-written, MATLAB-based programs.

Power spectral density was estimated using direct multi-taper estimators (10 Hz bandwidth, 3 tapers, 200 ms spectral window padded to double its length with zeros). To remove the slow frequency envelope, the LFP was whitened prior to spectral analysis. For whitening LFP signal was fitted with 2nd order autoregressive (AR) model (Mitra and Pesaran, 1999) and then the same filter coefficients were used across all channels and animals to remove the low frequency component accounted by the AR model. For assessment of the developmental profile of KA-Os, power spectral density in the beta or gamma band in CA3 pyramidal cell layer was determined as the peak power at peak frequency in power spectral density of whitened signal normalized to the baseline activity preceding kainate application.

**Figure 2:**
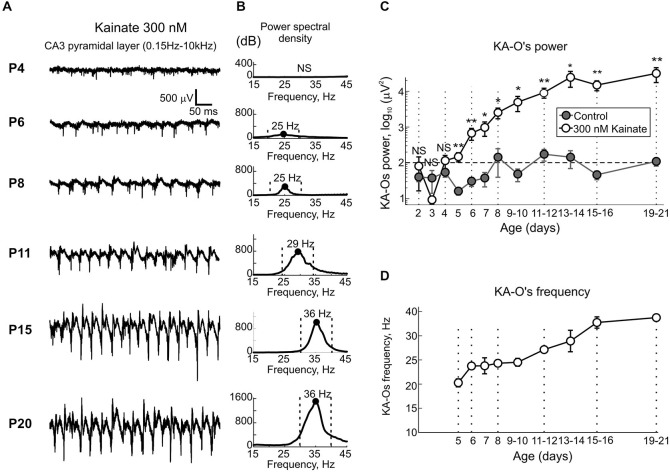
**Age-dependence of the kainate-induced oscillations in CA3 hippocampus. (A)** Example traces of full-band (0.15 Hz–10 kHz) extracellular field potential recordings from CA3 pyramidal cell layer in the presence of 300 nM—kainate in hippocampal slices obtained from animals of varying postnatal age. Note kainate inability to evoke oscillatory activity in slices obtained from P4 rat, kainate induced low-amplitude beta-activity at P6 and P8 and the progressive developmental increase in KA-Os’ amplitude and frequency. **(B)** Corresponding plots of the power spectral density decimated to the baseline activity prior to kainate application. Peak power is annotated (black dot) and the peak (±10 Hz) frequency range used to calculate the integral KA-O power is indicated with vertical dashed lines. Note absence of KA-Os at P4 (NS, non-significant) and a developmental increase in KA-Os’ power and frequency from beta to gamma frequency range through the period from P6 to P15. **(C,D)** Postnatal changes in the integral KA-Os’ power **(C)** and frequency **(D)**. Integral KA-Os’ power was calculated within peak ±10 Hz frequency band as shown on **(B)**. Note that before P5, local field potential (LFP) power in the presence of kainate does not significantly exceed the power of baseline activity (*gray*) and builds up to attain a steady level by the end of the first postnatal week. Note also that KA-Os are initially slow, lying within beta frequency range (20–25 Hz) but progressively increase in frequency to attain gamma frequency values by the end of the second postnatal week. **(C,D)** Pooled data from *n* = 119 slices obtained from *n* = 51 P2–21 animals. Error bars indicate SE. Hereafter *= *P* < 0.05, **= *P* < 0.01.

For circular statistics, instantaneous KA-Os’ phases were computed for the LFP recorded in the CA3 pyramidal cell layer. First, the dominant frequency was calculated in 200 ms length overlapped windows of LFP signal, further the LFP was filtered using Butterworth filter with band-pass in the range of dominant frequency peak ± 10 Hz as shown on Figure 2B. Second, instantaneous KA-Os’ phases were computed using Hilbert transformation of the narrowed band filtered signal. Third, the phase of each detected MUA spike within a KA-Os’ cycle was derived from the instantaneous KA-Os’ phase. Phase modulation of spikes in hippocampal CA3 pyramidal layer by gamma oscillations was determined using Rayleigh circular statistics. Group comparisons were done using parametric Watson-Williams multi-sample test and non parametric multi-sample test for equal medians. *P*-value of 0.05 was considered significant.

Current-source density (CSD) analysis across depth was used to eliminate volume conductance and localize synaptic currents. CSD was computed for each recording site according to differential scheme for second derivative and smoothed with a triangular kernel of length 4 (Freeman and Nicholson, 1975).

Instantaneous KA-Os’ amplitude was obtained through division of the KA-Os filtered signal by cosine of Hilbert transformation angle. Phase-amplitude coupling (PAC) analysis was performed using MATLAB toolbox of A. Onslow.

## Histology

Slices were fixed overnight in Paraformaldehyde (4%). After thorough rinsing in PBS, slices were mounted in fluoromount and coverslipped. Location of recording sites was identified by DiI staining of the electrodes.

### Drugs

All reagents were from Sigma (Sigma-Aldrich Inc., USA).

## Results

### Developmental Profile of KA-Os in CA3 Hippocampus

**Figure 3:**
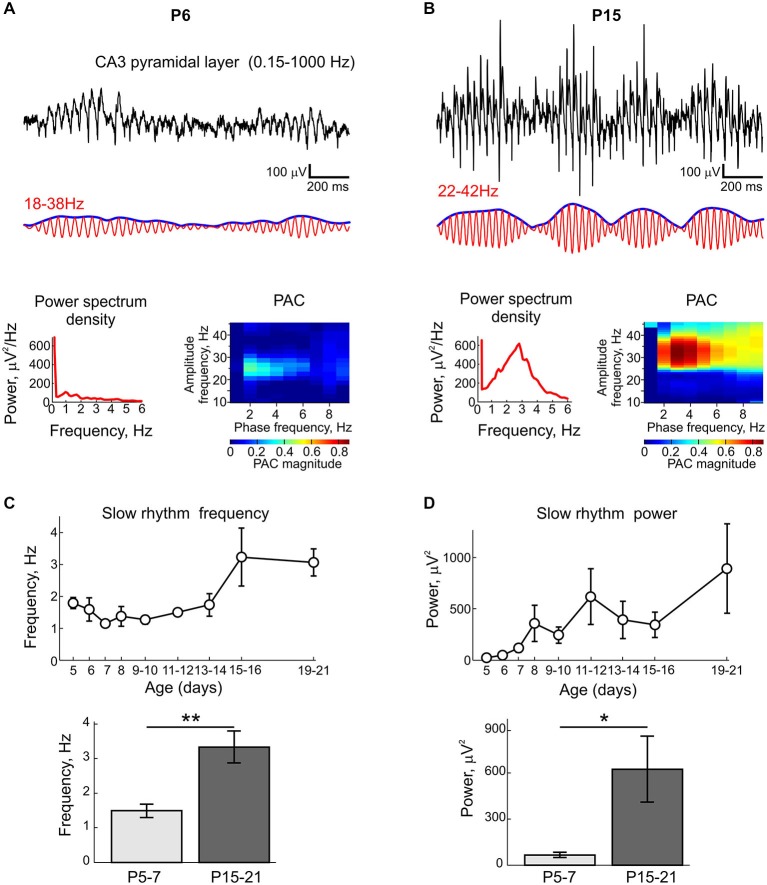
**Modulation of KA-Os by slow rhythms. (A,B)** Example traces of oscillatory activity recorded from CA3 pyramidal cell layer (black traces), band-pass filtered at beta **(A)** and gamma **(B)** frequency bands (red) and instantaneous KA-Os amplitude (blue) in slices from P6 **(A)** and P15 **(B)** rats. Below, corresponding power spectral densities and phase-amplitude coupling (PAC) color histograms. **(C,D)** Postnatal changes in slow rhythms frequency **(C)** and power **(D)**. Below, group data for P5–7 and P15–21 respective values. Pooled data from *n* = 35 slices.

In the present study, we used extracellular recordings of the LFP and MUA from CA3a region of the rat hippocampal slices in the interface chamber to characterize the postnatal development of kainate-induced gamma oscillations. In this aim, we recorded 119 slices from 51 P2–21 rats, with 6–14 slices per each postnatal day. Under control conditions, slices from P2–13 rats displayed spontaneous Giant Depolarizing Potentials (GDPs) as described previously (Ben-Ari et al., 1989; Khazipov et al., 2004). Application of kainate (100–500 nM) typically evoked an increase in GDPs’ frequency and a series of interictal—like events (not shown) (Khalilov et al., 1999) followed by steady-state KA-Os in an age-dependent manner (Figure 2). Up to postnatal age P5, despite of an increase of the ongoing spontaneous MUA, kainate in the range of concentrations from 100 nM to 1 μM failed to induce any detectable LFP oscillations whose power significantly exceeded baseline activity prior to application of kainate (*n* = 24 slices from P2–4 rats, Figure 2C). Starting from P5 onwards, kainate (300 nM) reliably evoked oscillations in CA3 pyramidal cell layer and their amplitude and frequency progressively increased with age (Figure 2A). Power spectrum density analysis showed that in P5–10 slices, KA-Os are characterized by peak frequency in beta range (mean, 24.3 ± 0.6 Hz; *n* = 48 slices from P5–10 rats; Figure 2B). These beta oscillations typically occurred in a spindle-shape bursts lasting for 0.5–1 s and they were weakly modulated by slower rhythms at 1–4 Hz as evidenced by PAC and instantaneous gamma amplitude analysis (Figure 3). Peak frequency values of KA-Os increased with age to attain gamma range values of 33.6 ± 1.1 Hz at P15–16 (*n* = 9 slices from P15–16 rats; Figures 2B,D). No further developmental increase in KA-Os frequency was observed at P19–21 (35.2 ± 0.8 Hz; *n* = 13 slices from P15–16 rats). Developmental increase in KA-Os frequency was paralleled with an increase in KA-Os amplitude (Figure 2A) and their modulation by slow rhythm (Figure 3). To estimate KA-Os’ power, we calculated the integral power in the frequency range of peak frequency ±10 Hz shown by vertical dashed lines on Figure 2B. As indicated above, in P2–4 slices LFP power in the presence of kainate did not significantly differ from the baseline activity (gray), and starting from P5 onwards KA-Os emerged and their power progressively increased with age to reach a plateau, by the end of the second postnatal week (*n* = 31; P13–21 slices; Figure 2C). KA-Os showed stability in frequency and power through continuous application of kainate. At P5, KA-Os frequency and power were of 18.1 ± 0.2 Hz and 293.1 ± 14.5 μV^2^ 10 min after kainate application and of 20.9 ± 0.4 Hz and 280.4 ± 12.4 μV^2^ 1 h after kainate application, respectively. At P15, KA-Os frequency and power were of 30.9 ± 1.6 Hz and KOs power were of 22.8 ± 1.7E3 μV^2^ 10 min after kainate application and of 30.1 ± 0.8 Hz and 18.3 ± 4.8E3 μV^2^ 1 h after application. Along with the developmental increase in KA-Os, the frequency and power of the slow rhythm modulating KA-Os also showed a developmental increase from 1.5 ± 0.2 Hz and 65 ± 17.4 μV^2^ at P5–7 to 3.3 ± 0.5 Hz and 623.6 ± 215.3 μV^2^ at P15–21, respectively (Figures 3C,D). Thus, kainate evokes fast oscillations in CA3 hippocampal slices commencing from P5 but not before, and KA-Os show developmental increase in power and frequency from beta to gamma values through a period from P5 to the end of the second postnatal week.

### Developmental Increase in CA3 Unit Synchronization by KA-Os

**Figure 4:**
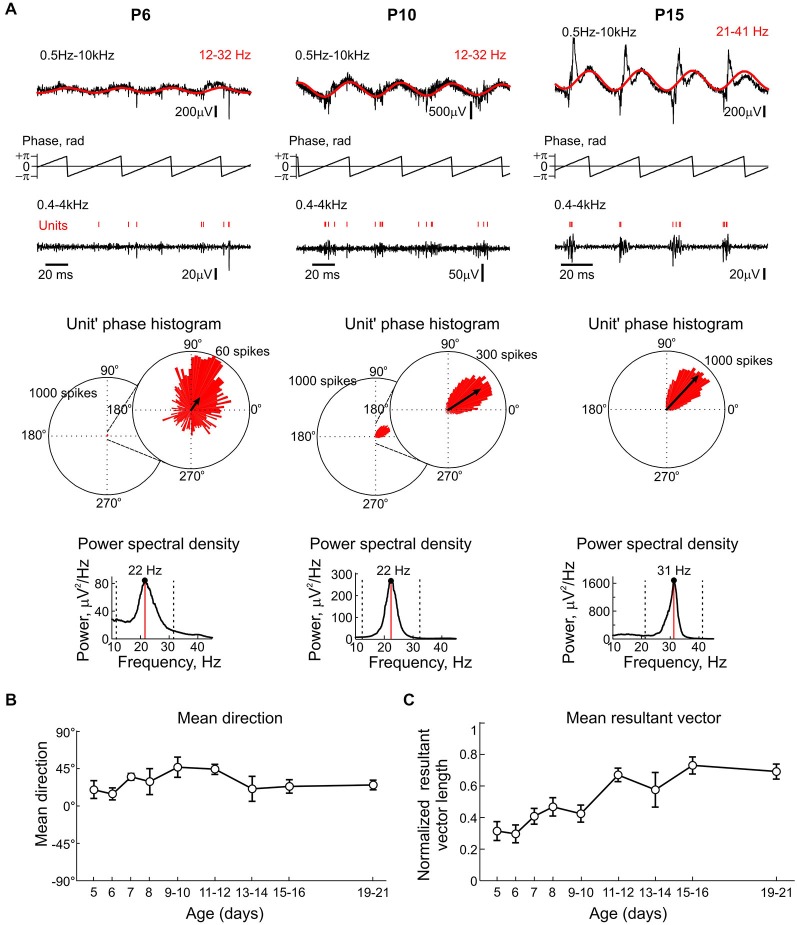
**Phase modulation of CA3 units by KA-Os through the postnatal development. (A)** Example full-band traces of LFP in the CA3 pyramidal cell layer at three different ages (*black*) with overlaid KA-Os- filtered trace (*red*, filter borders are indicated at the top-right). Middle traces show instantaneous KA-Os phases computed using Hilbert transformation. Bottom traces show corresponded multiple unit activity (MUA)-filtered trace with units indicated by vertical red bars. Below, circular unit’ phase histograms at the scale of 1000 spikes and rescaled histograms at 60 and 300 spikes at P6 and P10, respectively. Histograms were built from 1845, 6388, 17202 spikes recorded in P6, P10 and P15 rat slices, respectively during 100 s recording sessions. Corresponding LFP power spectral densities are shown on insets. **(B,C)** Summary plots of the age-dependence of the mean direction **(B)** and mean resultant vector amplitudes **(C). (B,C)** Pooled data from *n* = 81 slices obtained from *n* = 37 P5–21 animals.

We further addressed the developmental changes in neuronal synchronization by KA-Os. LFP signals from CA3 pyramidal cell layer were first filtered with ±10 Hz-wide filter centered at the peak of KA-Os frequency (Figure 4A, red traces overlaying the LFP signals), instantaneous KA-Os’ phase was computed using Hilbert transformation of the filtered signal and the phase of each detected MUA spike within a KA-Os’ cycle was derived from the instantaneous KA-Os’ phase (Figure 4A). Rayleigh circular statistics revealed relatively poor phase modulation of CA3 MUA by the early beta KA-Os and its increase with age. Rayleigh resultant vector increased from 0.31 ± 0.04 at P5–6 (*n* = 12 slices) to 0.73 ± 0.05 at P15–16 (*n* = 10 slices; Figure 4C). The phase preference of MUA to KA-Os remained unchanged during the postnatal development (Figure 4B). Thus, early beta KA-Os exhibit relatively poorly synchronized CA3 MUA, and with development there is an increase in synchronization level of CA3 MUA by KA-Os.

### Current-Source Density Analysis

**Figure 5:**
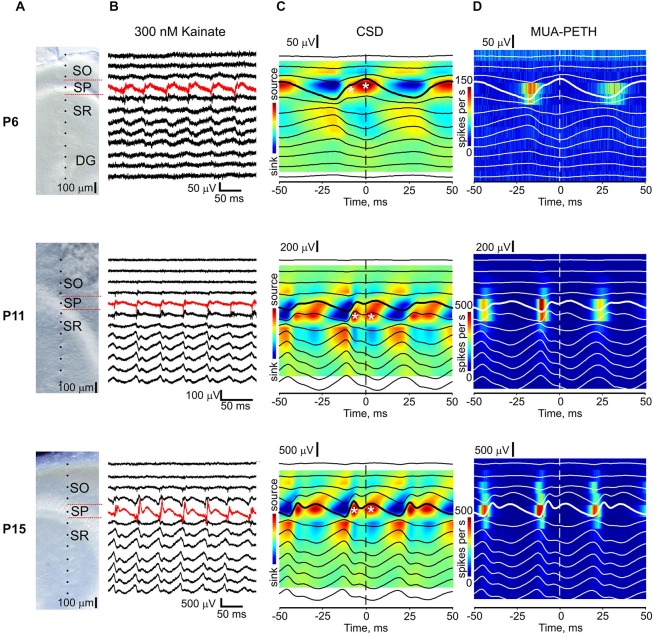
**Current-source density (CSD) profile of the early KA-Os. (A)** Microphotographs of CA3 region of the recorded slices at different ages. Dots indicate the location of the extracellular sites of 16-shank silicone probes. SO, stratum oriens; SP, stratum pyramidale; SR, stratum radiatum; DG, granular cell layer of gentate gyrus. **(B)** Example traces of LFP recordings at the electrodes shown on **(A)** during KA-Os. Red traces show the recordings from CA3 pyramidal cell layer. **(C)**, KA-Os’ peak triggered average LFP (black traces, bold signal is from CA3 pyramidal cell layer) overlaid on CSD of KA-Os. Time = 0 corresponds to the KA-Os’ peak. White asterisks indicate two sources associated with the peaks of KA-Os at the pyramidal cell layer. **(D)** KA-Os’ peak triggered average MUA peri-event time histograms. **(C,D)** average of 2090 (P6), 18163 (P11) and 6282 (P15) KA-O cycles.

CSD analysis of KA-Os was performed using 16-electrode recordings with a 100 μm pitch in *n* = 27 slices from P6–21 rats. Electrodes were placed across CA3 pyramidal cell layer to trace the activity from stratum oriens, pyramidale and radiatum as shown in Figures 5A,B. CSD analysis revealed alternating sinks and sources during KA-Os (Figure 5C) similar to those observed during cholinergic evoked gamma oscillations (Mann et al., 2005; Oren et al., 2010). Sinks associated with KA-Os troughs were located in CA3 pyramidal cell layer and corresponding sources were located in stratum oriens and radiatum. These troughs-related sinks were associated with a sharp increase in MUA as illustrated in Figure 5D and likely involved somatic currents through the voltage-gated sodium channels (Oren et al., 2010). KA-Os’ peaks were typically associated with biphasic sources (marked by asterisks on Figure 5C) also located in the pyramidal cell layer. The first source was a brief event curtailing the MUA discharge, it was more prominent in older animals and is likely to reflect pyramidal cell after-hyperpolarization mediated by potassium currents. It may also involve a passive source generated by recurrent collateral EPSCs which sink is located in stratum radiatum (Figures 7, 9). The second longer source was also associated with an inhibition of MUA and its decay paralleled with a gradual increase in MUA, which culminated during the next trough of KA-Os. This component likely involves perisomatic GABAergic currents (see also Figure 7) (Mann et al., 2005; Oren et al., 2010). This second source exhibited a longer duration in slices from P5–8 rats. Such an observation is in keeping with the longer duration of GABAergic synaptic currents in immature CA3 pyramidal cells (Taketo and Yoshioka, 2000) and may also reflect less synchronous recruitment of interneurons in the young animals. Thus, the CSD profile of both early beta and gamma KA-Os are characterized by alternating sinks and sources likely arising from perisomatic inhibition-based oscillations alternating with population discharge related sinks (Csicsvari et al., 2003; Khazipov and Holmes, 2003; Mann et al., 2005; Gulyás et al., 2010; Oren et al., 2010). This result suggests that the generative mechanisms of KA-Os are fundamentally similar across all ages examined in this current study despite of a difference in the peak oscillation frequency.

### Synaptic Correlates of KA-Os

**Figure 6:**
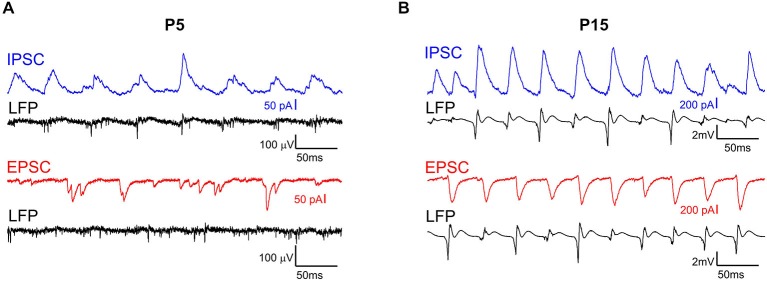
**Whole-cell patch-clamp recording of CA3 pyramidal cells during KA-Os**. Example traces of spontaneous activity recorded from CA3 pyramidal cells in voltage clamp mode at holding potential 0 mV (blue) and −50 mV (red) in hippocampal slices prepared from P5 **(A)** and P15 **(B)** rats in the presence of 300 nM—kainate. Below are shown simultaneous LFP recordings (black) from CA3 pyramidal cell layer in vicinity of the patched cell.

**Figure 7:**
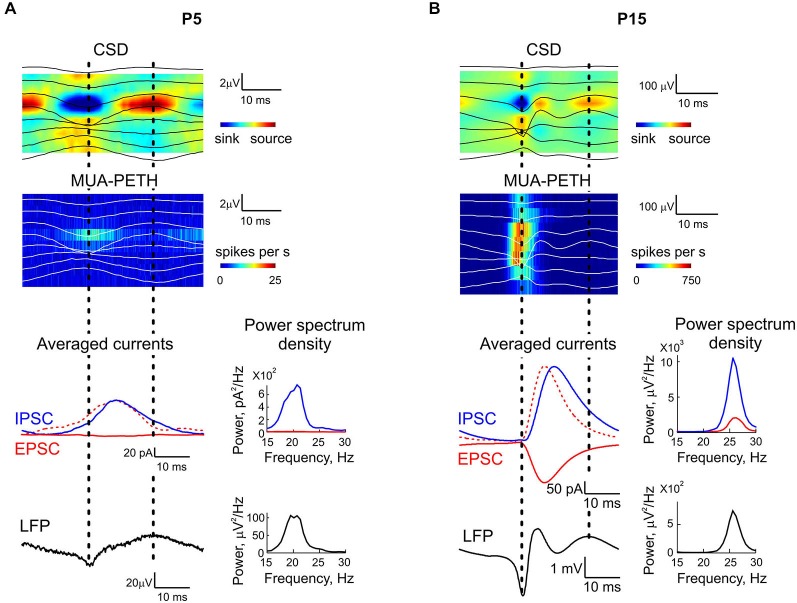
**IPSCs and EPSCs in relation to KA-Os cycles**. Representative cases of CSD and MUA-PETH averaged by KA-Os troughs in pyramidal cell layer with corresponding averaged IPSCs (blue line) and EPSCs (red solid line) recorded from CA3 pyramidal cells in a P5 **(A)** and P15 **(B)** rat hippocampal slices. Red dashed lines show inverted EPSCs scaled to the peak IPSC amplitude. Note a few milliseconds delay of IPSCs from EPSCs. Corresponding power spectrum densities of IPSC, EPSC and LFP are shown on the right.

**Figure 8:**
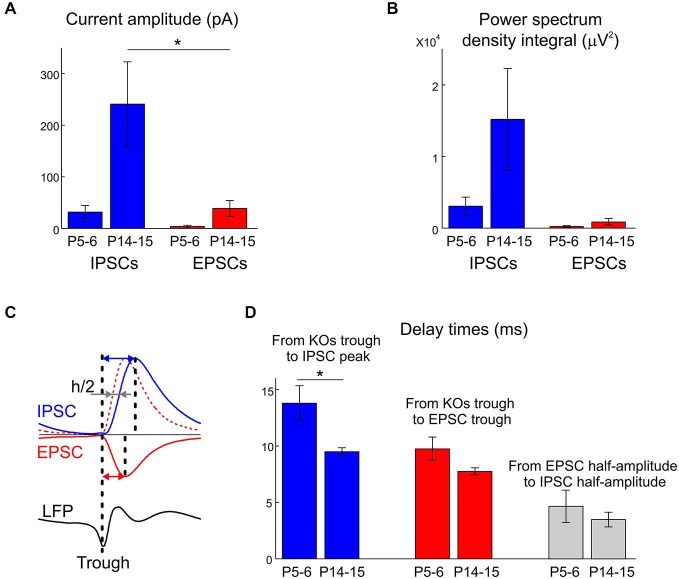
**Group data statistics on the developmental changes in synaptic contributions to KA-Os. (A)** Amplitudes of IPSCs and EPSCs averaged by KA-Os’ troughs in P5–6 and P14–15 age groups. **(B)** PSD integrals of IPSCs and EPSCs within the KA-Os frequency band in the two age groups. **(C)** Calculation of delays. Blue and red arrows show the times from the KA-Os troughs to IPSCs and EPSCs peaks; grey arrows show half-rise time delay between EPSCs and IPSCs. **(D)** Summary of the developmental changes in delays measured as shown on panel **(C). (A–D)** Pooled data from 12 cells. Error bars indicate SE.

We further explored synaptic correlates of KA-Os using whole-cell recordings from CA3 pyramidal cells and concomitant LFP recordings with 16-shank silicone probes in two age groups: P5–6 and P14–15. GABAergic and glutamatergic postsynaptic currents (IPSCs and EPSCs, respectively) were voltage separated during recordings with low chloride concentration in the pipette solution (IPSCs reversal potential was of −50 mV). We found that IPSCs and EPSCs were modulated by KA-Os in both age groups; however, synaptic contributions to oscillations were age-dependent and differed for IPSCs and EPSCs (Figure 6). In the young group (P5–6) KA-O troughs’ averaged IPCSs attained maximal value of 31.4 ± 12.4 pA at 13.8 ± 1.5 ms after KA-Os’ troughs and the mean integral PSD of IPCSs was of 3034.8 ± 1290.1 pA^2^ (*n* = 6 cells; Figures 7A, 8A). EPSCs showed much weaker KA-Os’ modulation with the mean amplitude of 3.4 ± 2.4 pA attained at 9.8 ± 1 ms after the KA-Os’ trough and with the mean EPSCs integral KA-Os PSD of 211.7 ± 124.2 pA^2^ (*n* = 6 cells; Figures 6A, 7A).

In the older age group (P14–15) both GABAergic and glutamatergic contributions to KA-Os significantly increased, with the mean IPSCs’ amplitude and integral PSD of 31.4 ± 12.4 pA and 3034.8 ± 1290.1 pA^2^, respectively, and with the mean EPSCs’ amplitude and integral PSD of 240.6 ± 82 pA and 16464.3 ± 7918.1 pA^2^, respectively (*n* = 6 cells; Figures 6B, 7B, 8A,B). Peak values of EPSCs and IPSCs were attained at a delay of 7.8 ± 0.3 ms and 13.8 ± 1.5 ms after the KA-Os’ trough, respectively (Figures 7B, 8D). In both age groups EPSCs preceded IPSCs by 4.7 ± 1.4 ms at P5–6 (*n* = 4 cells) and 3.5 ± 0.6 ms at P14–15 (*n* = 5 cells; Figure 8C), suggesting that rhythmic activation of GABAergic neurons during KA-Os involves an excitatory drive from the pyramidal cells thus indicating pyramidal-interneuron network gamma (PING) mechanism of KA-Os (Whittington et al., 2011). In addition to the age-dependent differences in synaptic contributions to KA-Os we also found that CA3 pyramidal cells from the younger age group (P5–6) were less depolarized in the presence of 300 nM kainate (−47.4 ± 5.1 mV, *n* = 5 cells) than P14–15 neurons (−26.6 ± 5.4 mV, *n* = 8 cells) that is in agreement with a developmental increase in the kainate-evoked currents in CA3 pyramidal cells (Khalilov et al., 1999).

### Developmental KA-Os Rely on GABA(A) Receptor Mediated Inhibition

**Figure 9:**
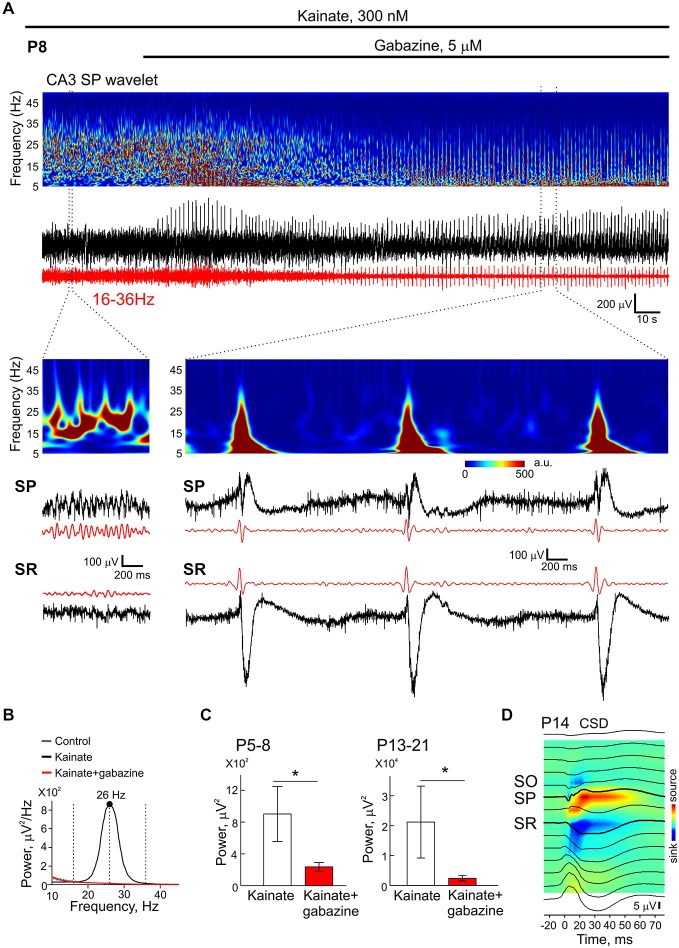
**Blockade of GABA(A) receptors suppresses KA-Os. (A)** Example traces of LFP in CA3 pyramidal cell layer of a P8 rat in the presence of 300 nM—kainate and after addition of the GABA(A) receptor antagonist gabazine (5 μM). Corresponding wavelet analysis is shown on the plots above traces. KA-Os’ filtered traces are shown in red. Extended traces show concomitant recordings from the pyramidal layer (SP) and stratum radiatum (SR). **(B)** Power spectral density of KA-Os presented on (A) before (black) and after (red) addition of gabazine. **(C)** Effect of gabazine on integral KA-Os’ power in two age groups. Pooled data from 8 (P5–8) and 4 (P15–21) slices. **(D)** Averaged epileptiform events (*n* = 70 events) recorded in the presence of KA (300 nM) and gabazine (5 μM) across CA3 layers and corresponding CSD. Note sinks in the stratum radiatum and oriens.

In keeping with the pivotal role of perisomatic GABAergic inhibition in generation of fast network oscillations in CA3 of the hippocampus, we further examined the effect of the selective GABA(A) receptor antagonist gabazine on KA-Os at varying ages. Specifically we examined the action of the antagonist on beta KA-Os at P5–8 and on gamma KA-Os at P15–21. In both age groups, gabazine largely suppressed KA-Os as evidenced by wavelet and power spectral analysis (Figures 9A–C) indicating that GABA(A) receptor mediated mechanisms are as important in generation of beta KA-Os in the immature rat slices as in gamma-rhythmogenesis in adult animals (Fisahn et al., 1998; Csicsvari et al., 2003; Khazipov and Holmes, 2003; Mann et al., 2005; Gulyás et al., 2010; Oren et al., 2010). During perfusion with gabazine in the presence of kainate, recurrent epileptiform events emerged in all slices studied. These epileptiform events typically lasted for 200 ms, recurred at a rate of about 0.5–1 Hz and were characterized by MUA bursts in pyramidal cell layer and massive sinks in stratum radiatum and oriens (Figure 9D). CSD-profile of these epileptiform events demonstrated that they are clearly distinct from KA-Os and indicated the major contribution of the local recurrent collateral synapses to their generation.

### Blockade of Depolarizing GABA does not Affect KA-Os

**Figure 10:**
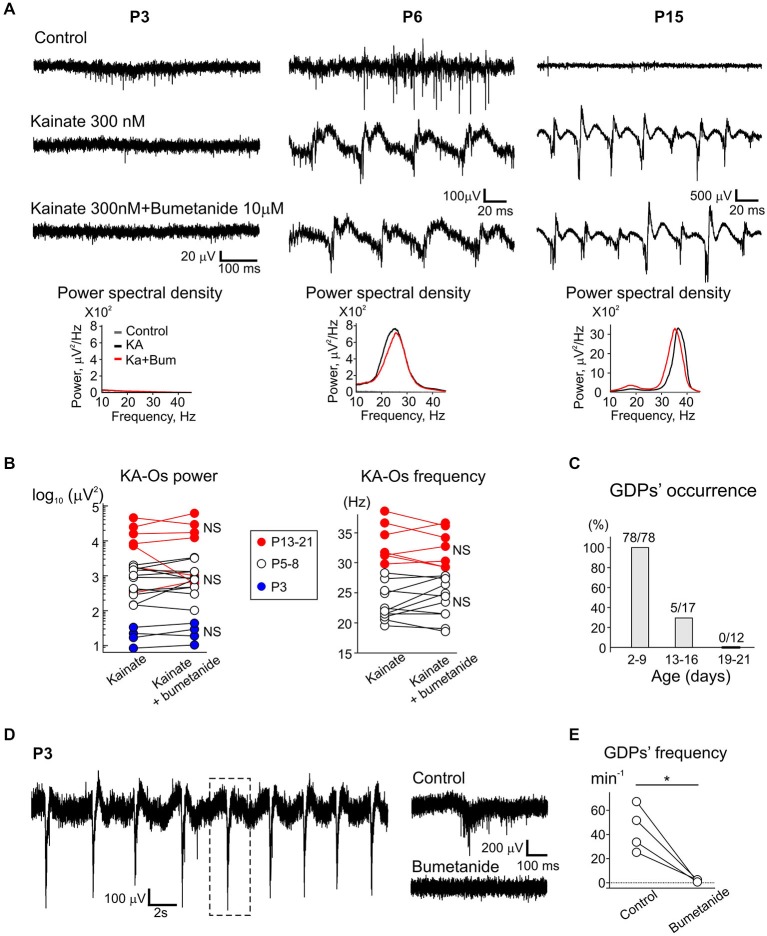
**Blockade of NKCC1 co-transporter with bumetanide does not affect KA-Os. (A)** Example traces of the activity recorded from CA3 pyramidal cell layer in control conditions (upper traces), in the presence of 300 nM-kainate (middle traces) and in the presence of 300 nM-kainate and 10 μM-bumetanide (lower traces) in P3, P6 and P15 slices. Corresponding power spectral densities are shown on insets below. Example traces at P3 and P6 show GDPs in control condition. **(B)** Effect of bumetanide on integral KA-Os’ power and frequency (pooled data from 4 (P3), 8 (P5–8) and 4 (P15–21) slices). At P3, bumetanide was pre-applied 10 min prior to combined application with kainate and the responses were compared with the responses evoked by kainate only; in older animals, bumetanide was applied in the presence of kainate. **(C)** Occurrence of GDPs in slices for three age groups. **(D)** Effect of bumetanide on GDPs. An example trace of the activity in CA3 pyramidal cell layer in control conditions. Dot rectangle presents border of extended trace shown in left. Right, bottom: example trace of CA3 pyramidal activity in the presence of bumetanide. **(E)** Summary plot of the effect of bumetanide on GDPs (pooled data from 4 slices, P3).

The depolarizing action of GABA on the immature neurons has been suggested as one of the factors limiting inhibition-based gamma rhythmogenesis in the developing circuits (Traub et al., 1997; Doischer et al., 2008; Whittington et al., 2011). Therefore the developmental excitatory effects of GABA may explain the lack of KA-Os in slices obtained from animals up to P5. This depolarizing effect on immature CA3 pyramidal cells can be suppressed pharmacologically by the NKCC1 antagonist bumetanide (Yamada et al., 2004; Dzhala et al., 2005; Sipilä et al., 2006; Tyzio et al., 2006; Valeeva et al., 2010, 2013; Minlebaev et al., 2013). Using this selective antagonist we examined two distinct hypotheses. First, is the lack of KA-Os during the first postnatal days is due to the presence of depolarizing GABA? Second, is depolarizing GABA critical for the maintenance of KA-Os in the older animals? We found that at P3, bumetanide completely suppressed GDPs in keeping with results of previous studies (Dzhala et al., 2005; Sipilä et al., 2006; Valeeva et al., 2010) (Figures 10C–E). Yet, kainate was unable to induce any oscillatory activity in the presence of bumetanide at P3 (Figures 10A,B). Bumetanide (10 μM) also affected neither power nor frequency of beta and gamma KA-Os in P5–8 and P13–21 age groups (Figures 10A,B). Thus, the delayed emergence and the developmental changes in KA-Os unlikely depend on the depolarizing actions of GABA on the neonatal hippocampal neurons. At higher concentrations bumetanide also blocks chloride extruder KCC2 which is known to be rapidly upregulated in P5–7 rat hippocampus after kainate-evoked hyperactivity (Khirug et al., 2010). We found that bumetanide at higher concentrations did not significantly affect KA-Os which frequency and power in presence of 50 μM bumetanide attained 92.4 + 5.1% and 105.4 + 1.8% of the control values, respectively.

### Sharp-Waves are Evoked by Kainate Starting from P10

**Figure 11:**
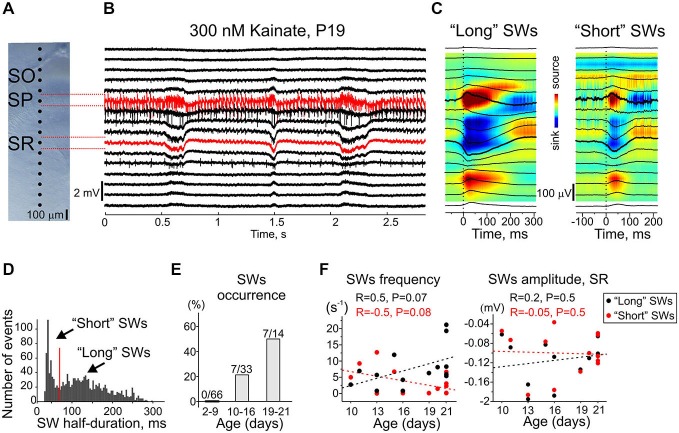
**Development of kainate-induced sharp-waves. (A)** Microphotographs of CA3 region of the recorded slice. Dots indicate the location of the extracellular sites of 16-shank silicone probe. SO, stratum oriens; SP, stratum pyramidale; SR, stratum radiatum. **(B)** Example trace of LFP recordings at the electrodes shown on **(A)** during SWs. Red traces show the recordings from CA3 pyramidal cell layer and stratum radiatum. **(C)** SWs’ front triggered average LFP (black traces) overlaid on CSD. Time = 0 corresponds to the SWs’ front. **(D)** Half-duration histogram of SWs. **(E)** SWs’ occurrence for three age groups (66 P2–9 slices, 33 P10–16 slices and 14 P19–21 slices). **(F)** Frequency (left) and amplitude in stratum radiatum (right) of short (red dots) and long (black dots) SWs. Dashed lines show linear regression of pooled data (47 slices P10–21 with SWs). R, linear regression coefficient and *P*-values are shown at the top.

In addition to fast network oscillations, kainate also evoked sharp-wave-like events (SWs) in some slices and their emergence was age-dependent (Figure 11). Although transient population events were often observed at the onset of kainate application at different ages, SWs were never observed during continuous application of kainate in <P10 slices (*n* = 66 slices from P2–9 rats). SWs were first observed at P10 (1 slice of 3), and their occurrence increased with age to attain 50% occurrence at P19–21 (*n* = 7 slices of 14) (Figure 11E). SWs were characterized by firing of CA3 units and robust sinks in stratum radiatum, and by CSD profile (Figure 11C) similar to that of the epileptifrom events observed in the presence of gabazine (Figure 9). SWs could be clearly separated in two classes based on their duration: short SWs of 30–70 ms half-duration and long SWs of 71–300 ms half-duration (Figure 11D). CSD profiles of both classes of events were similar (Figure 11C). The tendency to decrease of short and increase of long SWs frequency was observed with age without a change in their amplitude (Figure 11F). KA-Os were modulated by SWs and their power was strongly reduced following SWs. Kainate-induced SWs were often associated with high-frequency 85–150 Hz ripple-like oscillations in the pyramidal cell layer. These results are coherent with the developmental profile of the high-frequency oscillations emerging by the end of the second postnatal week in the rat hippocampus *in vivo* (Leinekugel et al., 2002; Buhl and Buzsáki, 2005; Mohns and Blumberg, 2008).

## Discussion

The main finding of the present study is that kainate-induced, inhibition based fast network oscillations emerge in CA3 subfield of the rat hippocampus commencing at P5 in the form of beta oscillations and develop to highly coherent gamma oscillations through the second postnatal week. The developmental profile of KA-Os matches the development of beta-gamma oscillations *in vivo* and the developmental changes in perisomatic GABAergic inhibition.

Early in development, GABA exerts depolarizing and excitatory actions on neurons and supports various types of slow (0.1–0.33 Hz) rhythmic activity such as recurrent GDPs in hippocampal slices and intact hippocampus *in vitro* (Ben-Ari et al., 1989; Khazipov et al., 2004; Valeeva et al., 2013). GDPs are eliminated after blockade of depolarizing and excitatory actions of GABA with NKCC1 antagonist bumetainde (Dzhala et al., 2005; Sipilä et al., 2006; Valeeva et al., 2010). Because GABA(A) receptor mediated inhibition is instrumental in generation of fast oscillations, depolarizing action of GABA could be a cause of the absence of fast oscillations in the immature cortex as hypothesized previously (Ben-Ari, 2002; Lahtinen et al., 2002; Sauer and Bartos, 2010). In the present study, the pre-application of bumetanide and subsequent addition of KA did not enable expression of KA-Os in slices from P3 rats, and the antagonism of NKCC1 with this compound had no effect on KA-Os in older animals. These results indicate that developmental changes in GABA polarity play little role in the development of fast oscillations and absence of KA-Os until P5 likely reflects immaturity of perisomatic-targeting GABAergic circuitry rather than depolarizing actions of GABA during the early postnatal period.

Indeed, the onset of KA-Os at around P5 and their developmental changes between P5 and P13–14 well match the maturation of perisomatic inhibition, which is known to be instrumental in generation of fast oscillations. Incorporation of the perisomatic-projecting basket cells into the network and development of fast-spiking features proceeds from the end of the first postnatal week through the first postnatal month (Du et al., 1996; Chattopadhyaya et al., 2004; Daw et al., 2007; Huang et al., 2007; Doischer et al., 2008; Okaty et al., 2009; Wang and Gao, 2010; Cossart, 2011; Goldberg et al., 2011; Pangratz-Fuehrer and Hestrin, 2011; Le and Monyer, 2013; Yang et al., 2014). During the first postnatal days, the pioneer GABAergic synapses established on pyramidal cells are located on dendrites but not on pyramidal cell somata (Dupuy and Houser, 1996; Tyzio et al., 1999). The earliest functional, presumably perisomatic synapses between the fast spiking interneurons and stellate cells in L4 barrel cortex were first reported at P4, and these were rare (<10% connection probability) and had low conductance; developmental surge in connectivity and conductance was found to occur between P3–5 and P7–9 (Daw et al., 2007). Accordingly, contribution of interneurons to generation of the early gamma oscillations in barrel cortex *in vivo* was not observed before P5 (Minlebaev et al., 2011; Khazipov et al., 2013). Similarly, hippocampal parvalbumin expressing basket cells are characterized by slow action potential duration and propagation time, release period, low frequency firing and make few low conductance and slow decay constant synapses on granule cells and other basket cells at P6 (Doischer et al., 2008). In addition, excitatory inputs to basket cells during this early period remain weak and essentially subthreshold (Daw et al., 2007; Doischer et al., 2008). This is in keeping with an absence of the feedforward inhibition, which is also mediated by the perisomatic-projecting parvalbumin interneurons in L4 neocortex until P6–7 *in vitro* and until P4–5 *in vivo* (Daw et al., 2007; Minlebaev et al., 2011). Although the exact timing of development of perisomatic inhibition in CA3 hippocampus remains to be determined, basing on the data obtained in other cortical regions we suggest that the onset of CA3 KA-Os, which we observed at around P5 is determined by delayed recruitment of CA3 perisomatic inhibition. Similarly, the developmental increase in KA-Os frequency, power and unit synchronization that we observed between P5 and P13–14 likely reflects developmental changes in perisomatic inhibition. This developmental period is characterized by a number of critical features: (i) a dramatic increase in hippocampal basket cell—principal neuron and basket cell—basket cell connectivity; (ii) an enforcement of glutamatergic inputs onto basket cells; (iii) the formation of electrical synapses between basket cells; (iv) an increase in unitary conductance combined with an acceleration of the decay in GABAergic connections made by basket cells; and (v) the acquisition of fast spiking features (decrease in action potential duration, conduction and release period) by basket cells (Du et al., 1996; Taketo and Yoshioka, 2000; Chattopadhyaya et al., 2004; Daw et al., 2007; Huang et al., 2007; Doischer et al., 2008; Okaty et al., 2009; Wang and Gao, 2010; Goldberg et al., 2011; Pangratz-Fuehrer and Hestrin, 2011; Le and Monyer, 2013; Yang et al., 2014). The developmental model of the inhibition-based gamma rhythmogenesis based on the developmental changes in perisomatic inhibition predicts that inhibition-based gamma oscillations should emerge in the rodent hippocampus by the end of the first postnatal week, and that they should be initially slow and poorly coherent (Doischer et al., 2008). The results of this present study provide direct experimental evidence that support the predictions derived from the model and *in vivo* data (Lahtinen et al., 2002; Leinekugel et al., 2002; Mohns and Blumberg, 2008).

## Author Contributions

RK and MOC conceived the project and designed experiments. VT performed the experiments. VT, MM and DS analyzed the data. RK and MOC wrote the paper.

## Conflict of Interest Statement

The authors declare that the research was conducted in the absence of any commercial or financial relationships that could be construed as a potential conflict of interest.

## References

[B1] Bartos M. Vida I. Jonas P. (2007). Synaptic mechanisms of synchronized gamma oscillations in inhibitory interneuron networks. Nat. Rev. Neurosci. 8, 45–56. 10.1038/nrn2044 17180162

[B2] Ben-Ari Y. (2002). Excitatory actions of GABA during development: the nature of the nurture. Nat. Rev. Neurosci. 3, 728–739. 10.1038/nrn920 12209121

[B3] Ben-Ari Y. Cherubini E. Corradetti R. Gaiarsa J.-L. (1989). Giant synaptic potentials in immature rat CA3 hippocampal neurones. J. Physiol. 416, 303–325. 10.1113/jphysiol.1989.sp017762 2575165 PMC1189216

[B4] Ben-Ari Y. Gaiarsa J. L. Tyzio R. Khazipov R. (2007). GABA: a pioneer transmitter that excites immature neurons and generates primitive oscillations. Physiol Rev. 87, 1215–1284. 10.1152/physrev.00017.2006 17928584

[B5] Buhl D. L. Buzsáki G. (2005). Developmental emergence of hippocampal fast-field “ripple” oscillations in the behaving rat pups. Neuroscience 134, 1423–1430. 10.1016/j.neuroscience.2005.05.030 16039793 PMC1851000

[B6] Buhl E. H. Tamás G. Fisahn A. (1998). Cholinergic activation and tonic excitation induce persistent gamma oscillations in mouse somatosensory cortex *in vitro*. J. Physiol. 513(Pt. 1), 117–126. 10.1111/j.1469-7793.1998.117by.x 9782163 PMC2231263

[B7] Buzsáki G. Draguhn A. (2004). Neuronal oscillations in cortical networks. Science 304, 1926–1929. 10.1126/science.1099745 15218136

[B8] Buzsáki G. Wang X. J. (2012). Mechanisms of gamma oscillations. Annu. Rev. Neurosci. 35, 203–225. 10.1146/annurev-neuro-062111-150444 22443509 PMC4049541

[B9] Chattopadhyaya B. Di Cristo G. Higashiyama H. Knott G. W. Kuhlman S. J. Welker E.. (2004). Experience and activity-dependent maturation of perisomatic GABAergic innervation in primary visual cortex during a postnatal critical period. J. Neurosci. 24, 9598–9611. 10.1523/jneurosci.1851-04.2004 15509747 PMC6730138

[B10] Cossart R. (2011). The maturation of cortical interneuron diversity: how multiple developmental journeys shape the emergence of proper network function. Curr. Opin. Neurobiol. 1, 160–168. 10.1016/j.conb.2010.10.003 21074988

[B11] Csicsvari J. Jamieson B. Wise K. D. Buzsáki G. (2003). Mechanisms of gamma oscillations in the hippocampus of the behaving rat. Neuron 37, 311–322. 10.1016/s0896-6273(02)01169-8 12546825

[B12] Daw M. I. Ashby M. C. Isaac J. T. (2007). Coordinated developmental recruitment of latent fast spiking interneurons in layer IV barrel cortex. Nat. Neurosci. 10, 453–461. 10.1038/nn1866 17351636

[B13] Doischer D. Hosp J. A. Yanagawa Y. Obata K. Jonas P. Vida I.. (2008). Postnatal differentiation of basket cells from slow to fast signaling devices. J. Neurosci. 28, 12956–12968. 10.1523/jneurosci.2890-08.2008 19036989 PMC6671784

[B14] Du J. Zhang L. Weiser M. Rudy B. McBain C. J. (1996). Developmental expression and functional characterization of the potassium-channel subunit Kv3.1b in parvalbumin-containing interneurons of the rat hippocampus. J. Neurosci. 16, 506–518. 8551335 10.1523/JNEUROSCI.16-02-00506.1996 PMC6578625

[B15] Dupuy S. T. Houser C. R. (1996). Prominent expression of two forms of glutamate decarboxylase in the embryonic and early postnatal rat hippocampal formation. J. Neurosci. 16, 6919–6932. 8824330 10.1523/JNEUROSCI.16-21-06919.1996 PMC6579262

[B16] Dzhala V. Khalilov I. Ben-Ari Y. Khazipov R. (2001). Neuronal mechanisms of the anoxia-induced network oscillations in the rat hippocampus *in vitro*. J. Physiol. 536, 521–531. 10.1111/j.1469-7793.2001.0521c.xd 11600686 PMC2278871

[B17] Dzhala V. I. Talos D. M. Sdrulla D. A. Brumback A. C. Mathews G. C. Benke T. A.. (2005). NKCC1 transporter facilitates seizures in the developing brain. Nat. Med. 11, 1205–1213. 10.1038/nm1301 16227993

[B18] Fisahn A. Contractor A. Traub R. D. Buhl E. H. Heinemann S. F. McBain C. J. (2004). Distinct roles for the kainate receptor subunits GluR5 and GluR6 in kainate-induced hippocampal gamma oscillations. J. Neurosci. 24, 9658–9668. 10.1523/jneurosci.2973-04.2004 15509753 PMC6730151

[B19] Fisahn A. Pike F. G. Buhl E. H. Paulsen O. (1998). Cholinergic induction of network oscillations at 40 Hz in the hippocampus *in vitro*. Nature 394, 186–189. 10.1038/28179 9671302

[B20] Freeman J. A. Nicholson C. (1975). Experimental optimization of current source-density technique for anuran cerebellum. J. Neurophysiol. 38, 369–382. 165272 10.1152/jn.1975.38.2.369

[B21] Fries P. (2009). Neuronal gamma-band synchronization as a fundamental process in cortical computation. Annu. Rev. Neurosci. 32, 209–224. 10.1146/annurev.neuro.051508.135603 19400723

[B22] Fries P. Neuenschwander S. Engel A. K. Goebel R. Singer W. (2001). Rapid feature selective neuronal synchronization through correlated latency shifting. Nat. Neurosci. 4, 194–200. 10.1038/84032 11175881

[B23] Goldberg E. M. Jeong H. Y. Kruglikov I. Tremblay R. Lazarenko R. M. Rudy B. (2011). Rapid developmental maturation of neocortical FS cell intrinsic excitability. Cereb. Cortex 21, 666–682. 10.1093/cercor/bhq138 20705896 PMC3041012

[B24] Gray C. M. Singer W. (1989). Stimulus-specific neuronal oscillations in orientation columns of cat visual cortex. Proc. Natl. Acad. Sci U S A 86, 1698–1702. 10.1073/pnas.86.5.1698 2922407 PMC286768

[B25] Gulyás A. I. Szabó G. G. Ulbert I. Holderith N. Monyer H. Erdélyi F.. (2010). Parvalbumin-containing fast-spiking basket cells generate the field potential oscillations induced by cholinergic receptor activation in the hippocampus. J. Neurosci. 30, 15134–15145. 10.1523/jneurosci.4104-10.2010 21068319 PMC3044880

[B26] Haggerty D. C. Glykos V. Adams N. E. LeBeau F. E. (2013). Bidirectional modulation of hippocampal gamma(20–80 Hz) frequency activity *in vitro* via alpha(alpha)- and beta(beta)-adrenergic receptors (AR). Neuroscience 253, 142–154. 10.1016/j.neuroscience.2013.08.028 23994151

[B27] Hájos N. Katona I. Naiem S. S. Mackie K. Ledent C. Mody I.. (2000). Cannabinoids inhibit hippocampal GABAergic transmission and network oscillations. Eur. J. Neurosci. 12, 3239–3249. 10.1046/j.1460-9568.2000.00217.x 10998107

[B28] Hormuzdi S. G. Pais I. LeBeau F. E. Towers S. K. Rozov A. Buhl E. H.. (2001). Impaired electrical signaling disrupts gamma frequency oscillations in connexin 36-deficient mice. Neuron 31, 487–495. 10.1016/s0896-6273(01)00387-7 11516404

[B29] Huang Z. J. Di Cristo G. Ango F. (2007). Development of GABA innervation in the cerebral and cerebellar cortices. Nat. Rev. Neurosci. 8, 673–686. 10.1038/nrn2188 17704810

[B30] Khalilov I. Dzhala V. Medina I. Leinekugel X. Melyan Z. Lamsa K.. (1999). Maturation of kainate-induced epileptiform activities in interconnected intact neonatal limbic structures *in vitro*. Eur. J. Neurosci. 11, 3468–3480. 10.1046/j.1460-9568.1999.00768.x 10564355

[B31] Khazipov R. Holmes G. L. (2003). Synchronization of kainate-induced epileptic activity via GABAergic inhibition in the superfused rat hippocampus *in vivo*. J. Neurosci. 23, 5337–5341. 12832559 10.1523/JNEUROSCI.23-12-05337.2003 PMC6741193

[B32] Khazipov R. Khalilov I. Tyzio R. Morozova E. Ben Ari Y. Holmes G. L. (2004). Developmental changes in GABAergic actions and seizure susceptibility in the rat hippocampus. Eur. J. Neurosci. 19, 590–600. 10.1111/j.0953-816x.2003.03152.x 14984409

[B33] Khazipov R. Leinekugel X. Khalilov I. Gaïarsa J.-L. Ben-Ari Y. (1997). Synchronization of GABAergic interneuronal network in CA3 subfield of neonatal rat hippocampal slices. J. Physiol. 498, 763–772. 10.1113/jphysiol.1997.sp021900 9051587 PMC1159192

[B34] Khazipov R. Minlebaev M. Valeeva G. (2013). Early gamma oscillations. Neuroscience 250, 240–252. 10.1016/j.neuroscience.2013.07.019 23872391

[B35] Khazipov R. Valeeva G. Khalilov I. (2015). Depolarizing GABA and developmental epilepsies. CNS Neurosci. Ther. 21, 83–91. 10.1111/cns.12353 25438879 PMC6495283

[B36] Khirug S. Ahmad F. Puskarjov M. Afzalov R. Kaila K. Blaesse P. (2010). A single seizure episode leads to rapid functional activation of KCC2 in the neonatal rat hippocampus. J. Neurosci. 30, 12028–12035. 10.1523/jneurosci.3154-10.2010 20826666 PMC6633538

[B37] Lahtinen H. Palva J. M. Sumanen S. Voipio J. Kaila K. Taira T. (2002). Postnatal development of rat hippocampal gamma rhythm *in vivo*. J. Neurophysiol. 88, 1469–1474. 10.1152/jn.00800.2001 12205167

[B38] Le M. C. Monyer H. (2013). GABAergic interneurons shape the functional maturation of the cortex. Neuron 77, 388–405. 10.1016/j.neuron.2013.01.011 23395369

[B39] Leinekugel X. Khazipov R. Cannon R. Hirase H. Ben Ari Y. Buzsáki G. (2002). Correlated bursts of activity in the neonatal hippocampus *in vivo*. Science 296, 2049–2052. 10.1126/science.1071111 12065842

[B40] Mann E. O. Paulsen O. (2007). Role of GABAergic inhibition in hippocampal network oscillations. Trends Neurosci. 30, 343–349. 10.1016/j.tins.2007.05.003 17532059

[B41] Mann E. O. Suckling J. M. Hajos N. Greenfield S. A. Paulsen O. (2005). Perisomatic feedback inhibition underlies cholinergically induced fast network oscillations in the rat hippocampus *in vitro*. Neuron 45, 105–117. 10.1016/j.neuron.2004.12.016 15629706

[B42] Minlebaev M. Colonnese M. Tsintsadze T. Sirota A. Khazipov R. (2011). Early gamma oscillations synchronize developing thalamus and cortex. Science 334, 226–229. 10.1126/science.1210574 21998388

[B43] Minlebaev M. Valeeva G. Tcheremiskine V. Coustillier G. Khazipov R. (2013). Cell-attached recordings of responses evoked by photorelease of GABA in the immature cortical neurons. Front. Cell. Neurosci. 7:83. 10.3389/fncel.2013.00083 23754981 PMC3668178

[B44] Mitra P. P. Pesaran B. (1999). Analysis of dynamic brain imaging data. Biophys. J. 76, 691–708. 10.1016/s0006-3495(99)77236-x 9929474 PMC1300074

[B45] Mohns E. J. Blumberg M. S. (2008). Synchronous bursts of neuronal activity in the developing hippocampus: modulation by active sleep and association with emerging gamma and theta rhythms. J. Neurosci. 28, 10134–10144. 10.1523/jneurosci.1967-08.2008 18829971 PMC2678192

[B46] Okaty B. W. Miller M. N. Sugino K. Hempel C. M. Nelson S. B. (2009). Transcriptional and electrophysiological maturation of neocortical fast-spiking GABAergic interneurons. J. Neurosci. 29, 7040–7052. 10.1523/jneurosci.0105-09.2009 19474331 PMC2749660

[B47] Oren I. Hájos N. Paulsen O. (2010). Identification of the current generator underlying cholinergically induced gamma frequency field potential oscillations in the hippocampal CA3 region. J. Physiol. 588, 785–797. 10.1113/jphysiol.2009.180851 20051494 PMC2834938

[B48] Pais I. Hormuzdi S. G. Monyer H. Traub R. D. Wood I. C. Buhl E. H.. (2003). Sharp wave-like activity in the hippocampus *in vitro* in mice lacking the gap junction protein connexin 36. J. Neurophysiol. 89, 2046–2054. 10.1152/jn.00549.2002 12686578

[B49] Pangratz-Fuehrer S. Hestrin S. (2011). Synaptogenesis of electrical and GABAergic synapses of fast-spiking inhibitory neurons in the neocortex. J. Neurosci. 31, 10767–10775. 10.1523/jneurosci.6655-10.2011 21795529 PMC3159030

[B50] Sakatani S. Seto-Ohshima A. Shinohara Y. Yamamoto Y. Yamamoto H. Itohara S.. (2008). Neural-activity-dependent release of S100B from astrocytes enhances kainate-induced gamma oscillations *in vivo*. J. Neurosci. 28, 10928–10936. 10.1523/jneurosci.3693-08.2008 18945900 PMC6671364

[B51] Sauer J. F. Bartos M. (2010). Recruitment of early postnatal parvalbumin-positive hippocampal interneurons by GABAergic excitation. J. Neurosci. 30, 110–115. 10.1523/JNEUROSCI.4125-09.2010 20053893 PMC6632526

[B52] Singer W. Gray C. M. (1995). Visual feature integration and the temporal correlation hypothesis. Annu. Rev. Neurosci. 18, 555–586. 10.1146/annurev.neuro.18.1.555 7605074

[B53] Sipilä S. T. Schuchmann S. Voipio J. Yamada J. Kaila K. (2006). The cation-chloride cotransporter NKCC1 promotes sharp waves in the neonatal rat hippocampus. J. Physiol. 573, 765–773. 10.1113/jphysiol.2006.107086 16644806 PMC1779742

[B54] Taketo M. Yoshioka T. (2000). Developmental change of GABA(A) receptor-mediated current in rat hippocampus. Neuroscience 96, 507–514. 10.1016/s0306-4522(99)00574-6 10717431

[B55] Traub R. D. Jefferys J. G. Whittington M. A. (1997). Simulation of gamma rhythms in networks of interneurons and pyramidalX cells. J. Comput. Neurosci. 4, 141–150. 10.1023/A:1008839312043 9154520

[B56] Tyzio R. Cossart R. Khalilov I. Minlebaev M. Hübner C. A. Represa A.. (2006). Maternal oxytocin triggers a transient inhibitory switch in GABA signaling in the fetal brain during delivery. Science 314, 1788–1792. 10.1126/science.1133212 17170309

[B57] Tyzio R. Minlebaev M. Rheims S. Ivanov A. Jorquera I. Holmes G. L.. (2008). Postnatal changes in somatic gamma-aminobutyric acid signalling in the rat hippocampus. Eur. J. Neurosci. 27, 2515–2528. 10.1111/j.1460-9568.2008.06234.x 18547241

[B58] Tyzio R. Represa A. Jorquera I. Ben-Ari Y. Gozlan H. Aniksztejn L. (1999). The establishment of GABAergic and glutamatergic synapses on CA1 pyramidal neurons is sequential and correlates with the development of the apical dendrite. J. Neurosci. 19, 10372–10382. 10575034 10.1523/JNEUROSCI.19-23-10372.1999 PMC6782402

[B59] Uhlhaas P. J. Roux F. Rodriguez E. Rotarska-Jagiela A. Singer W. (2010). Neural synchrony and the development of cortical networks. Trends Cogn. Sci. 14, 72–80. 10.1016/j.tics.2009.12.002 20080054

[B60] Valeeva G. Abdullin A. Tyzio R. Skorinkin A. Nikolski E. Ben-Ari Y.. (2010). Temporal coding at the immature depolarizing GABAergic synapse. Front. Cell. Neurosci. 4:17. 10.3389/fncel.2010.00017 20725525 PMC2914581

[B61] Valeeva G. Valiullina F. Khazipov R. (2013). Excitatory actions of GABA in the intact neonatal rodent hippocampus *in vitro*. Front. Cell. Neurosci. 7:20. 10.3389/fncel.2013.00020 23467988 PMC3587803

[B62] Wang X. J. (2010). Neurophysiological and computational principles of cortical rhythms in cognition. Physiol. Rev. 90, 1195–1268. 10.1152/physrev.00035.2008 20664082 PMC2923921

[B63] Wang H. X. Gao W. J. (2010). Development of calcium-permeable AMPA receptors and their correlation with NMDA receptors in fast-spiking interneurons of rat prefrontal cortex. J. Physiol. 588, 2823–2838. 10.1113/jphysiol.2010.187591 20547673 PMC2956901

[B64] Whittington M. A. Cunningham M. O. LeBeau F. E. Racca C. Traub R. D. (2011). Multiple origins of the cortical gamma rhythm. Dev. Neurobiol. 71, 92–106. 10.1002/dneu.20814 21154913

[B65] Yamada J. Okabe A. Toyoda H. Kilb W. Luhmann H. J. Fukuda A. (2004). Cl- uptake promoting depolarizing GABA actions in immature rat neocortical neurones is mediated by NKCC1. J. Physiol. 557, 829–841. 10.1113/jphysiol.2004.062471 15090604 PMC1665166

[B66] Yang J. M. Zhang J. Yu Y. Q. Duan S. Li X. M. (2014). Postnatal development of 2 microcircuits involving fast-spiking interneurons in the mouse prefrontal cortex. Cereb. Cortex 1, 98–109. 10.1093/cercor/bhs291 23042741

